# Dickkopf-1 as a Novel Predictor Is Associated with Risk Stratification by GRACE Risk Scores for Predictive Value in Patients with Acute Coronary Syndrome: A Retrospective Research

**DOI:** 10.1371/journal.pone.0054731

**Published:** 2013-01-24

**Authors:** Lin Wang, Xiao Bo Hu, Wei Zhang, Lin Di Wu, Yu Sheng Liu, Bo Hu, Cheng Long Bi, Yi Fei Chen, Xin Xin Liu, Cheng Ge, Yun Zhang, Mei Zhang

**Affiliations:** 1 The Key Laboratory of Cardiovascular Remodeling and Function Research, Chinese Ministry of Education and Chinese Ministry of Public Health, Shandong University Qilu Hospital, Jinan, Shandong, People’s Republic of China; 2 Shandong Provincial Hospital Affiliated to Shandong University, Jinan, Shandong, People’s Republic of China; 3 The Second Hospital of Shandong University, Jinan, Shandong, People’s Republic of China; University of Freiburg, Germany

## Abstract

**Objective:**

Dickkopf-1 (DKK-1), a major regulator of the Wnt pathway, plays an important role in cardiovascular disease. However, no study has evaluated the association of DKK-1 and acute coronary syndrome (ACS). We investigated this association and whether the Global Registry of Acute Coronary Events (GRACE) hospital-discharge risk score predicting major adverse cardiac events (MACE) can be improved by adding the DKK-1 value.

**Methods:**

We enrolled 291 patients (46 with ST-segment elevation myocardial infarction [STEMI] and 245 with non-ST elevated ACS [NSTE-ACS]) who were divided into groups by tertiles of baseline plasma DKK-1 level measured by ELISA. The GRACE risk score was calculated and predictive value alone and together with DKK-1 and/or high-sensitivity C-reactive protein (hs-CRP) level were assessed, respectively.

**Results:**

Compared with patients with NSTE-ACS, those with STEMI had higher plasma DKK-1 level at baseline (*P* = 0.006). Plasma DKK-1 level was correlated with hs-CRP level (*r* = 0.295, *P*<0.001) and was greater with high than intermediate or low GRACE scores (*P* = 0.002 and *P*<0.001, respectively). We found 44 (15.1%) MACEs during a median 2-year follow-up. DKK-1 levels were higher for patients with than without events (*P*<0.001). The rate of MACE increased with increasing DKK-1 level (*P*<0.001). The area under the receiver operating characteristic curve for GRACE score with MACE was 0.524 and improved to 0.791 with the addition of hs-CRP level, 0.775 with the addition of DKK-1 level and 0.847 with both values added.

**Conclusions:**

DKK-1 is an independent predictor of long-term MACE of patients with ACS. The long-term predictive ability of post-discharge GRACE score may be enhanced by adding DKK-1 level.

## Introduction

Accumulating evidence indicates that atherosclerosis is a chronic disease characterized by inflammation and lipid accumulation [1], [2]. Inammation is an important mechanism of atherosclerosis, atherosclerotic plaque progression, or even predisposing vulnerable plaque to rupture. Therefore, inammatory markers are predictors of recurrent events in ACS. Levels of plasma markers of inflammation such as CRP are elevated in acute coronary syndrome (ACS) [3].

Recent data point to a key role of the Wnt signaling pathway in the regulation of inflammation [4]. The Wnt pathway is regulated by multiple families of secreted antagonists, including soluble frizzled related receptors and dickkopfs (DKK); the best-studied of DKKs is DKK-1. Recent reports [5] showed increased expression of DKK-1 in advanced atherosclerotic plaque, and serum levels of DKK-1 gave prognostic information for patients with multiple myeloma and other malignancies, as well as osteoarthritis [6], [7], [8]. The inammatory process that underlines atherosclerosis is mediated by a multitude of cytokines and is unlikely to be totally reected by CRP level alone [9], [10], [11], [12].

No previous study has evaluated the association of DKK-1 and ACS with the Global Registry of Acute Coronary Events (GRACE) hospital-discharge risk scores predicting major adverse cardiac events (MACE), nor an association with MACE at 2-year follow-up. Hence, we sought to gain greater insight into the association of the inflammatory biomarkers DKK-1 and high-sensitivity CRP (hs-CRP) and baseline characteristics of patients with ACS to improve the predictive performance of the validated and well-performing GRACE risk scores.

## Methods

### Study Population

We included consecutive patients hospitalized in the Department of Cardiology of Qilu Hospital, Shandong University, from March 2008 to January 2010. Inclusion criteria were diagnosis of ACS, including ST-segment elevation myocardial infarction (STEMI) and non-ST elevated ACS (NSTE-ACS); all patients underwent coronary angiography. Exclusion criteria were valvular heart disease, severe arrhythmias, active hepatosis, malignant diseases, anemia and acute or chronic inflammatory diseases. The diagnosis of STEMI was typical chest pain with serum cardiac enzyme levels twice that of the upper level of normal or cardiac troponin I (cTnI) level ≥0.1 ng/ml, both with persistent electrocardiographic ST segment elevation >1 mm in 2 or more contiguous leads or newly occurred left bundle branch block. NSTE-ACS included non-STEMI (NSTEMI) and unstable angina (UA). The diagnosis of NSTEMI was angina or discomfort at rest with ST segment depression or transient elevation and/or prominent T-wave inversion, with cardiac enzyme levels twice that of the upper level of normal or cTnI ≥0.1 ng/ml. Patients with clinical features and/or electrocardiographic expression of NSTEMI but normal cardiac biomarker levels were diagnosed as having UA. The study protocol was approved by the Clinical Research Ethic Committee of Qilu Hospital, Medical Colledge of Shandong University. The study was in accordance with principles of Helsinki Declaration and all patients provided written informed consent.

### Laboratory Analysis

Blood samples were collected in EDTA-containing tubes and then centrifuged at 4°C. The collected plasma was stored in aliquots at −80°C. DKK-1 concentration was measured by use of an ELISA kit (R&D Systems, Minneapolis, USA). All laboratory data, including total cholesterol (TC), triglycerides (TG), high-density lipoprotein-cholesterol (HDL-C), low-density lipoprotein-cholesterol (LDL-C), blood glucose, uric acid level, creatinine level, creatinine kinase activity, and cTnI and hs-CRP levels were measured in the biochemical department of Qilu Hospital.

### Calculation of GRACE Risk Scores

The main principle of the GRACE risk score has been described elsewhere [13]. The variables required for calculation of the score include age, heart rate, systolic blood pressure, baseline creatinine level, history of congestive heart failure, in-hospital percutaneous coronary intervention, history of MI, ST-segment depression on admission electrocardiography (ECG) and elevated cardiac enzyme or marker levels.

In this study, we used single serum levels of cTnI >0.06 ng/ml as the elevated cardiac marker. ST-segment depression was defined as decreased ST segment≥0.5 mV below the isoelectric line in any ECG lead. The risk categories of GRACE score were divided into low, medium and high. For patients with STEMI, the risk scores were 27 to 99, 100 to 127, and 128 to 263, respectively [14]. For patients with non-ST elevated ACS (NSTE-ACS), the risk scores were 1 to 88, 89 to 118, and 119 to 263, respectively [14].

### Follow-up

Endpoints after discharge were MACEs, including sudden cardiac death, MI, percutaneous coronary intervention, coronary artery bypass grafting and recurrent unstable angina pectoris. Patients were followed up by researchers from Qilu Hospital, Shandong University.

### Statistical Analysis

All data were analyzed by use of SPSS v16.0 (SPSS Inc., Chicago, IL, USA). Numeric variables are expressed as mean±SD. Categorical variables are expressed as frequencies and percentages. Kolmogorov-Smirnov test was used to assess normal distribution of quantitative variables, with log transformation for non-normal distribution. Categorical data were compared by chi-square test or Fisher’s exact test as appropriate. Bivariate correlation was used for correlation analysis. One-way ANOVA was used for comparison of multiple groups [15]. Binary logistic regression was used to assess the independent association of DKK-1 level with MACE. Differences in the predictive values were estimated by comparing the area under the receiver-operating characteristic curve (ROC). The level of statistical significance was set at *P*<0.05.

## Results

### Baseline Characteristics of Study Subjects

**Figure 1:**
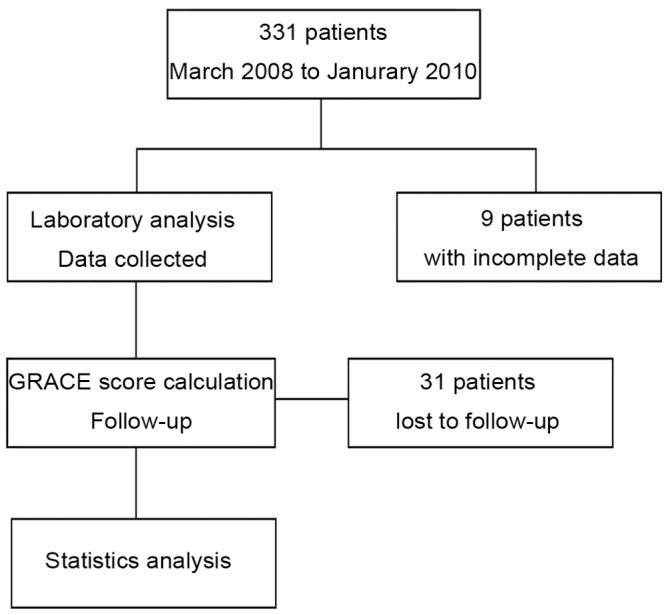
Flow chart of the study.

A total of 331 patients with ACS met the inclusion criteria, and we had complete data for 322. At the end of the study, data for 291 patients (193 males, 66.3%) with complete follow-up data were analyzed, including 46 with STEMI and 245 with NSTE-ACS (63 with NSTEMI, 182 with UA), and 68% of our patients underwent percutaneous coronary intervention. The flow chart of data in the study is presented in **Figure 1**. The demographic and clinical characteristics of patients grouped by tertiles of baseline DKK-1 level are in **Table 1**. All data were obtained within 24 hr after admission. Patients with high DKK-1 levels were older and had higher blood glucose and hs-CRP concentrations than others. DKK-1 level did not differ by coronary artery status.

**Table 1:** Baseline Characteristics of Patients with Acute Coronary Syndrome (ACS) By Tertiles of Dickkopf-1 (DKK-1) Level.

	DKK-1 level tertile			
Variables	T1 (n = 97)	T2 (n = 97)	T3 (n = 97)	*P* value
Age (yr)	55±10	56±10	59±10	**0.025**
Male, no. (%)	67 (69.1)	67 (69.1)	59 (60.8)	0.374
Body mass index (kg/m^2^)	26.2±3.44	26.6±3.19	26.4±6.35	0.848
Hypertension, no. (%)	64 (66)	62 (63.9)	56 (57.7)	0.524
SBP (mmHg)	132±15	132±16	130±13	0.432
DBP (mmHg)	78±11	76±11	75±10	0.317
Diabetes, no. (%)	25 (25.8)	22 (22.7)	30 (30.9)	0.421
Smoker, no. (%)	51 (52.6)	46 (47.4)	44 (45.4)	0.585
Total cholesterol (mmol/L)	4.59±0.87	4.68±0.83	4.73±0.84	0.493
Triglyceride (mmol/L)	1.66±0.55	1.71±0.57	1.81±0.59	0.180
HDL-cholesterol (mmol/L)	1.19±0.28	1.18±0.25	1.18±0.26	0.922
LDL-cholesterol (mmol/L)	2.69±0.68	2.62±0.63	2.70±0.60	0.659
Blood glucose (mmol/L)	6.18±0.90	6.25±0.87	6.58±0.94	**0.005**
Cr (µmol/L)	86.0±7.9	87.9±9.8	87.6±9.0	0.274
CK (µmol/L)	73.8±39.9	99.7±110.8	124.5±192.1	0.058
cTnI (ng/ml)	1.73±8.82	0.52±1.69	1.22±3.54	0.450
Uric acid (µmol/L)	315±45	316±48	312±44	0.836
Hs-CRP (pg/ml)	1.47±1.33	2.03±1.74	2.54±1.81	**<0.001**
PLT (10^9^/L)	220±64	225±60	232±67	0.424
Medical treatment, no. (%)				
ACE inhibitors	41 (42.3)	41 (42.3)	35 (36.1)	0.665
Beta blockers	69 (71.1)	68 (70.1)	74 (76.3)	0.109
Aspirin	96 (99.0)	95 (97.9)	97 (100)	0.999
CCB	13 (13.4)	10 (10.3)	15 (15.5)	0.299
Clopidogrel	66 (68.0)	65 (67.0)	64 (66.0)	0.384
Statins	80 (82.5)	70 (72.2)	84 (86.6)	0.730
Cardiovascular disease, no. vessels involved, no. (%)				0.964
1 vessel	37 (38.1)	34 (35.1)	34 (35.1)	
2 vessels	32 (33.0)	33 (34.0)	36 (37.1)	
3 vessels	28 (28.9)	30 (30.9)	27 (27.8)	
Stenosis degree, no. (%)				0.986
50–75%	41 (42.3)	42 (43.3)	42 (43.3)	
≥75%	56 (57.7)	55 (56.7)	55 (56.7)	

Data are mean±SD unless indicated. SBP, systolic blood pressure; DBP, diastolic blood pressure; HDL, high density lipoprotein; LDL, low density lipoprotein; Cr, creatinine; CK, creatinine kinase; cTnI, cardiac troponin I; hsCRP, high-sensitivity C-reactive protein level; ACE, angiotensin-converting enzyme; CCB, calcium channel blocker.

### Plasma Level of DKK-1 in Patients with ACS

**Figure 2:**
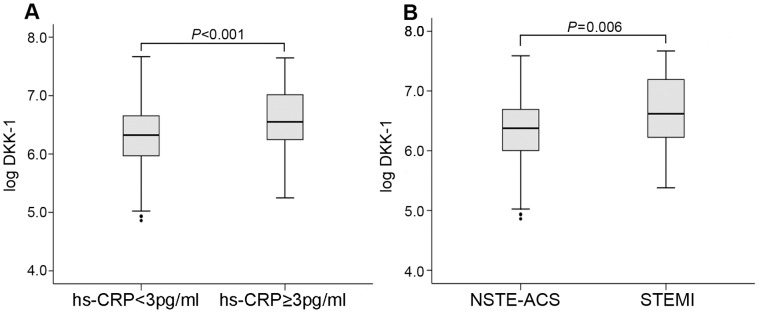
Log DKK-1 with ACS patients. (**A**) Relation of log DKK-1 level and high-sensitivity C-reactive protein (hs-CRP) level in ACS patients (*P*<0.001). The cutoff point for hs-CRP level was 3 pg/ml. (**B**) Log DKK-1 level in different ACS groups.

Median plasma DKK-1 level was 713 pg/ml (range 129–2139 pg/ml). DKK-1 levels were correlated with hs-CRP level (*r* = 0.295, P<0.001, **Fig. 2A**). DKK-1 was significantly higher in patients with STEMI than those with NSTE-ACS at baseline (*P* = 0.006, **Fig. 2B**). DKK-1 level did not differ between patients with NSTEMI and those with UA (*P*>0.05).

### Baseline Characteristics of ACS Patients During Follow-up

**Figure 3:**
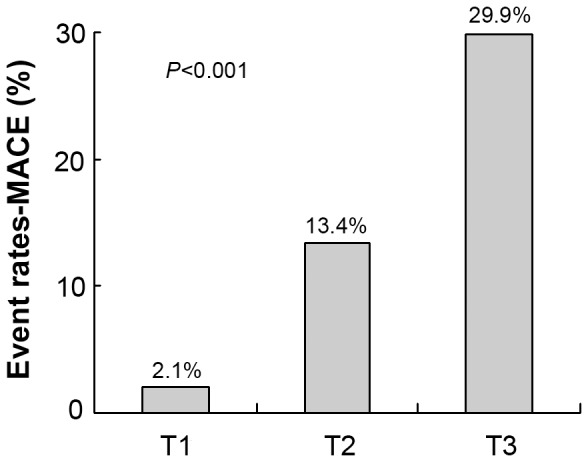
Association of tertiles of DKK-1 level and major adverse coronary events (MACE) (*P*<0.001).

We found 44 (15.1%) MACEs during a median 2-year follow-up: 4 sudden cardiac death, 30 unstable angina pectoris, 3 revascularization and 7 rehospitalization. Clinical data of patients by follow-up results are in **Table 2**. The status of coronary artery did not influence prognosis. Compared to patients without events, those with events were older, and hypertension and diabetes were more frequent. The groups differed in levels of TC (*P*<0.001), TG (*P = *0.014), LDL-C (*P = *0.028), HDL-C, blood glucose and hs-CRP (all *P*≤0.001). The event rates of MACE by DKK-1 tertile were higher for T3 than both T1 and T2 (*P*<0.001; **Fig. 3**).

**Table 2:** Baseline Characteristics of Patients With and Without Major Adverse Coronary Events.

Variables	With MACE (n = 44)	Without MACE (n = 247)	*P* value
Age (yr)	60±10	56±10	**0.039**
Male, no. (%)	30 (68.2)	163 (66.0)	0.777
Body mass index (kg/m^2^)	26.0±3.1	26.5±4.9	0.569
Hypertension, no. (%)	37 (84.1)	145 (58.7)	**0.001**
SBP (mmHg)	131±12	131±15	0.865
DBP (mmHg)	75±10	77±11	0.327
Diabetes, no. (%)	23 (52.3)	54 (21.9)	**0.001**
Smoker, no. (%)	27 (61.4)	114 (46.2)	0.063
Total cholesterol level (mmol/L)	5.09±0.67	4.59±0.85	**<0.001**
Triglycerides level (mmol/L)	1.92±0.47	1.69±0.58	**0.014**
HDL-cholesterol level (mmol/L)	1.07±0.16	1.21±0.27	**<0.001**
LDL-cholesterol level (mmol/L)	2.87±0.57	2.64±0.64	**0.028**
Blood glucose (mmol/L)	6.76±1.00	6.26±0.88	**0.001**
Cr (µmol/L)	87.7±8.86	87.1±9.00	0.694
CK activity (µmol/L)	121.3±186	95.3±118	0.258
cTnI level (ng/ml)	0.68±2.1	1.26±6.0	0.580
Uric acid level (µmol/L)	317±40	314±46	0.690
Hs-CRP level (pg/ml)	3.44±1.38	1.76±1.62	**<0.001**
DKK-1 level (pg/ml)	1064±486	641±364	**<0.001**
Cardiovascular disease, no. vessels involved, no. (%)			0.131
1 vessel	10 (22.7)	95 (38.5)	
2 vessels	19 (43.2)	82 (33.2)	
3 vessels	15 (34.1)	70 (28.3)	
Stenosis degree, no. (%)			0.338
50–75%	16 (36.4)	109 (44.1)	
≥75%	28 (63.6)	138 (55.9)	

### Associations of DKK-1 and Risk Stratification by GRACE Score

**Figure 4:**
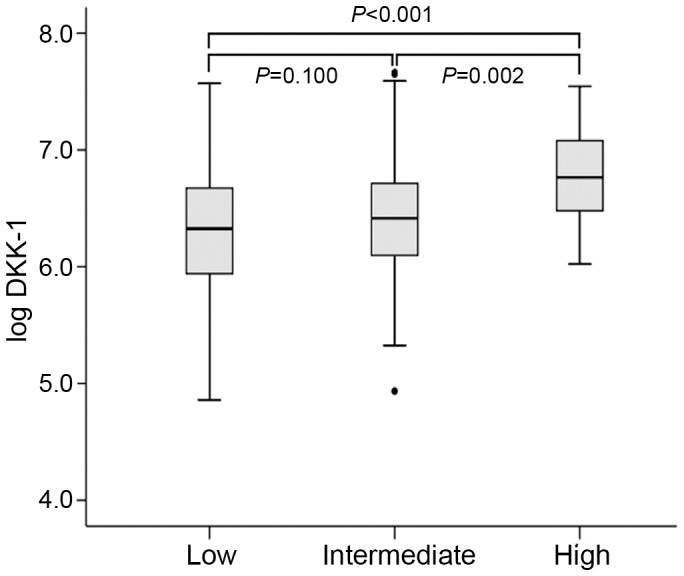
Log DKK-1 in patients with ACS in 3 subgroups by GRACE score. Data are median (range).

The medium concentrations of DKK-1 were 642, 718 and 959 pg/ml for low, intermediate and high GRACE category, respectively (**Fig. 4**). The concentrations of DKK-1 were elevated with high-risk than intermediate- or low-risk GRACE score (*P* = 0.002 and *P*<0.001). DKK-1 levels were higher but not significantly with intermediate than low risk (*P* = 0.100).

The median GRACE risk score was 88 (range 38–149) for the whole population, 108 (63–149) for patients with STEMI, 84 (38–148) for patients with NSTE-ACS, 89 (46–140) for patients with MACE and 88 (38–149) for patients without MACE. The GRACE scores were significantly higher for patients with STEMI than NSTE-ACS at baseline (*P*<0.001). MACE and non-MACE groups did not differ in GRACE scores (*P* = 0.570).

### DKK-1 is an Independent Predictor of Long-term MACE for Patients with ACS

After adjustment for cardiovascular risk factors, binary logistic regression revealed a significant association of DKK-1 and hs-CRP levels and MACE for ACS patients (OR 8.451, 95% CI 3.176–22.487, *P*<0.001; 1.395, 1.072–1.815, *P* = 0.013, respectively, **Table 3**).

**Table 3:** Binary Logistic Regression Analysis of Cardiovascular Risk Predictors for Patients with ACS.

Variables	OR	95% CI	*P* value
Age	1.047	0.993–1.103	0.090
Sex	0.487	0.135–1.760	0.272
Body mass index	0.872	0.745–1.021	0.089
Hypertension	0.167	0.048–0.577	**0.005**
Diabetes	0.370	0.137–1.003	0.051
Smoker	0.514	0.155–1.698	0.275
Hypercholesterolemia	2.971	1.383–6.385	**0.005**
Triglyceride level	0.805	0.339–1.912	0.623
LDL-C level	1.411	0.565–3.522	0.461
HDL-C level	0.093	0.009–0.977	**0.048**
Hs-CRP level	1.395	1.072–1.815	**0.013**
DKK-1 level	8.451	3.176–22.487	**<0.001**

OR, odds ratio; 95% CI, 95% confidence interval.

### DKK-1 has Better Prognostic Value for Patients with ACS

**Figure 5:**
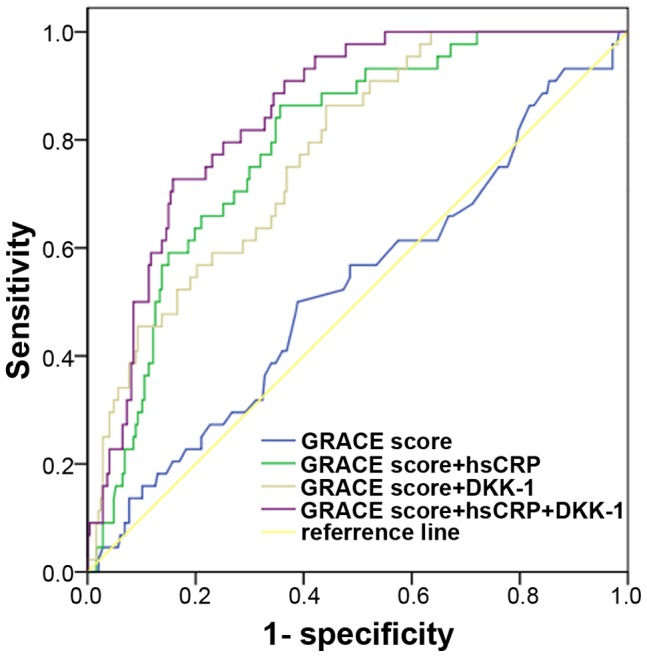
Receiver operating characteristic curve analysis of predictive models for ACS patients. The sensitivity and specificity for each model was 56.8% and 51.4%; 84.1% and 64.4%; 75.0% and 63.2%; 81.8% and 71.7%.

A 3-step process was used for ROC analysis: GRACE score alone, GRACE score with DKK-1 or hs-CRP level, and GRACE score with both biomarkers. The GRACE score alone was a poor predictor of MACE (with area under the ROC [AUC] 0.524). With the addition of DKK-1 level, the AUC was increased to 0.775 and to 0.791 with hs-CRP level. With both biomarkers added, the AUC was significantly increased to 0.847, and the sensitivity of this model in evaluating prognosis was 81.8%, with specificity 71.7% (**Fig. 5**).

## Discussion

DKK-1, as a major regulator of the Wnt pathway, plays a key role in cardiovascular disease. We investigated the association of DKK-1 in ACS and whether the GRACE hospital-discharge risk score for MACE could be improved by adding the DKK-1 value. We also investigated an association of DKK-1 level and MACE at 2-year follow-up. Plasma DKK-1 level at baseline was higher for patients with than without STEMI and was correlated with hs-CRP level. Plasma DKK-1 level was higher with high than intermediate or low GRACE scores and was higher for patients with than without MACE. The AUC for GRACE score predicting MACE was best with both hs-CRP and DKK-1 levels added. Plasma levels of DKK-1 may be useful for identifying and for long-term prediction for patients with ACS at high risk of MACE, especially when combined with hs-CRP for the GRACE score.

Numerous epidemiology studies have indicated the role of inflammation in atherosclerotic plaques and an association of circulating inflammatory markers such as CRP or interleukin-6 and severity of cardiac events in ACS [12], [16]. Abnormal Wnt signaling is associated with many human diseases and plays a distinct role in inflammation and immunity [17]. The Wnt pathways are regulated by multiple families of secreted antagonists, including soluble frizzled-related receptors and DKKs, the best-studied being DKK-1. DKK-1 has been implicated in cancer, brain ischemia, and bone disease [6], [7], [8], [18]; previous studies have shown a close association of serum levels of DKK-1 and atherosclerotic diseases such as premature myocardial infarction [19] or ischemic cerebrovascular disease [20]. The increased expression of DKK-1 in advanced carotid plaques enhancing the inflammatory interaction between platelets and endothelial cells might drive the inflammatory loop [5], Overexpression of DKK-1 was found in macrophages and endothelial cells, and immunostaining of thrombus material from the site of plaque rupture showed strong immunoreactivity in platelet aggregates.

As with previous findings [5], [19] we found plasma levels of DKK-1 greater in patients with STEMI than NSTE-ACS, and plasma levels of DKK-1 positively correlated with hs-CRP level. Similar to hs-CRP, DKK-1 might be a novel inflammatory biomarker in peripheral blood, with high levels correlated with atherosclerotic plaque destabilization or even rupture. Serum levels of DKK-1 might be useful for identifying or as a long-term predictive factor for patients with ACS at high risk of MACE [21], [22]. To further elucidate this issue, binary logistic regression analysis revealed that levels of TC, hs-CRP and DKK-1 were all independent risk factors for ACS patients, and DKK-1 was the strongest biochemical indicator.

Risk stratification of clinical events is an essential part of disease management, and the risk scoring system we adopted here (GRACE) represents the most widely used and validated risk scoring schemes for patients with ACS [23], [24]. GRACE scores for the prediction of various MACE, including death and re-infarction, have been well verified at various follow-up times and designated as low, intermediate and high [25]. We compared the capacity of these scores to predict the risk of events [26], [27] and found that the GRACE scores alone in our study did not have good performance. This finding might be influenced by mild disease status of our patients, such as the absence of abnormal creatinine level, which might impact the performance of GRACE score. Moreover, no inflammatory biomarkers were taken into account for calculating the GRACE score. The complexity between coronary instability and inflammation underlines the importance of biomarkers that might be useful in helping identifying high-risk patients from those classified as low-risk by GRACE score [28]. When we reanalyzed increased DKK-1 level in ACS patients with GRACE risk scores to MACE composite endpoints at a median of 2 years of follow-up, the predictive performance of the GRACE score was improved [23]. The level of DKK-1 was higher with than without MACE and was higher with high than intermediate or low GRACE scores, with no significant difference between intermediate and low scores. Increased level of DKK-1 may imply more serious coronary atherosclerosis and high-risk or vulnerable coronary plaque in patients with ACS. These data are in accordance with the report by Ueland et al. of the increased expression of DKK-1 in advanced atherosclerotic plaques. Our findings indicated that DKK-1 might be released into circulation in advanced atherosclerosis, atherosclerotic plaque destabilization or even rupture. This finding may explain the additive value of DKK-1 in improving the predictive ability of GRACE scores in our study. Another finding of the present study was that the prediction performance was significantly clarified by hs-CRP and DKK-1 level and their combination to the model. Thus, the combination of plasma levels of both hs-CRP and DKK-1 to GRACE scores was more valuable to predict cardiac events of patients with ACS at high risk of MACE [28].

Overall, a major discrepancy exists in the prognostic values of different biomarkers. Such a discrepancy underlines the complex and heterogeneous patterns linking coronary instability, biomarkers, and the point of their measurement, therapeutic strategies, and outcomes in the wide spectrum of patients with ACS.

### Study Limitations

Because of its exclusion criteria, our trial studied a select group of patients that might not reect the general population. As well, the clinical relevance of plaque stability observed with plasma DKK-1 level requires a larger sample size. Finally, this study might not fully represent disease progression elsewhere. Thus, the findings need to be validated by prospective large-sized population-based studies.

### Conclusions

The long-term predictive ability of the GRACE hospital-discharge risk score may be enhanced by adding DKK-1 level. DKK-1 has independent predictive value for long-term MACE of patients with ACS.
